# Terminology matters: is the International Association for the Study of Pain definition of pain fully satisfactory for fetuses, neonates, and infants?

**DOI:** 10.3389/fpain.2024.1369945

**Published:** 2024-05-16

**Authors:** M. E. Canepa, L. Raffini, L. A. Ramenghi

**Affiliations:** ^1^ Department of Neuroscience, Ophthalmology, Genetics and Mother-Child Health, University of Genoa, Genoa, Italy; ^2^ Department of Political and International Science (DISPI), University of Genoa, Genoa, Italy; ^3^ Neonatal Intensive Care Unit and Mother Child Division Chief, IRCCS Giannina Gaslini, Genoa, Italy

**Keywords:** pain, fetus, neonate, analgesia, neurodevelopment, sedation, pain memory, pain response

## 1 Introduction—the International Association for the Study of Pain definitions: potential inconsistencies

Could pain be well described with words? In 1979, the International Association for the Study of Pain (IASP) stated that pain was “an unpleasant sensory and emotional experience associated with actual or potential tissue damage or described in terms of such damage”.

In addition in 1979, IASP further explain with further notes that: “Pain is always subjective. Each individual learns the application of the word through experiences related to injury in early life. Biologists recognize that those stimuli which cause pain are liable to damage tissue”. This sentence is challenging or even impossible to be applied to fetuses and newborns, although focusing on the importance of early life experiences was a laudable insight.

Only in 2018, IASP revised the 1979 definition with an international task force and described pain as “an unpleasant sensory and emotional experience associated with or resembling that associated with actual or potential tissue damage”. Interestingly, the key change is “resembling associated with” following the previous incipit “an unpleasant sensory and emotional experience associated with”.

In the last definition few more notes from IASP were added, clarifying many aspects related to verbal patients but not improving the understanding of “pain in early life”.

1. Pain is always a personal experience that is influenced to varying degrees by biological, psychological, and social factors.
2. Pain and nociception are different phenomena. Pain cannot be inferred solely from activity in sensory neurons.
3. Through their life experiences, individuals learn the concept of pain.
4. A person's report of an experience as pain should be respected
5. Although pain usually serves an adaptive role, it may have adverse effects on function and social and psychological well-being.
6. Verbal description is only one of several behaviors to express pain; inability to communicate does not negate the possibility that a human or a nonhuman animal experiences pain.

In our opinion this definition with the explicative notes definitions failed to add significant improvements for pain experiences early in life. Bioethically, the presence or absence of fetal pain was used as an argument to set against or for the legalization of abortion (1). To understand pain-related issues better in neonatal and prenatal medicine, this political and often appearing as religious debate must be set aside from the historical path, and new scientific discoveries should be reviewed (2) in terms of physiological, behavioral and neural “signs of pain”.

## 2 The “fetal pain paradox” from the 1980s until birth

As fetuses and neonates are immature beings, they may be deemed incapable of judging their pain perception (3) in the context of the International Association for the Study of Pain definition (4). Furthermore, the notion that the cerebral cortex of fetuses and neonates is immature has dominated the scientific community in the 1980s–1990s (5–7). Due to this immaturity, fetuses were considered incapable of perceiving and reacting to pain until the third trimester of pregnancy in the 2000s (8). Ten years later, this incapability was denied by Sekulic and Bellieni, respectively suggesting that “the pain inhibition mechanisms are not sufficiently developed during intrauterine development leading to increased intensity of pain in the fetus” and the arise of pain as “a neuroadaptive phenomenon that emerges at about 20–22 weeks” (9, 10). But it was highlighted that fetuses can perceive and react to pain via the cortical subplate structures as early as 12 weeks of gestation (1, 3, 11, 12). The “fetal pain paradox” described by Thill (13) states that even below <24-week fetuses and preterm babies born present the same pain items (immature cortex, active and functional subplate, facial expression of pain in response to noxious stimuli, fight-or-flight stress in response to noxious stimuli, and body movement in response to noxious stimuli), pain management in preterm babies is deemed as an important clinical task, while fetuses are still not sufficiently and universally considered capable of perceiving pain (13).

## 3 More than pain memory, pain response and perception (nociception), sedation, and analgesia

Many researchers dislike the fact that the word “memory” is related to pain, although it remains unquestionable that early pain experiences are hard to live at any age, and certainly, these nociceptive experiences modify the way of reacting to subsequent painful experiences, as brilliantly shown by Anna Taddio in the Lancet. In this study, she showed that babies who underwent circumcision without adequate sedation cried much longer and were more difficult to console when exposed to injections for vaccination in the following months compared to their peers who were adequately sedated when receiving circumcisions as neonates (14). Analgesia is difficult in the neonatal age, in particular for normal babies exposed to noxious stimuli (i.e., heel pricks) as drugs cannot be given outside a neonatal intensive care units and, in addition, there is evidence that these drugs in sick babies are detrimental for brain development.

In early life, oral glucose can be considered a first analgesic drug, as its taste reduces nociceptive reaction (15). While sucking and tasting sucrose, neonates react less vigorously and cry much less during heel-prick procedures compared to that in controls. These effects are similar in premature infants, who are exposed to prolonged noxious stimuli (16). The positive effect of sweet taste was already discovered in the *De Rerum Natura*, as babies were exposed to the taste of honey to decrease the discomfort of nauseating medications (17). There are no doubts nociceptive reaction is acutely reduced during exposure to painful procedures, however, in a recent review reported that “oral sweet-tasting solutions should be used judiciously to mitigate behavioral responses to mild painful procedures” in neonates as long-term effects remain unexplored (18); moreover, these do not seem to be effective in attenuating the negative effects of pain on brain development (19). Additionally, a few effective drugs, such as midazolam, were discovered to be toxic for hippocampal development and neurodevelopmental outcomes (20) as this drug was associated with “macro- and microstructural alteration in hippocampal development and poorer outcomes consistent with hippocampal dysmaturation”, with a lower hippocampal volume generated by midazolam doses (20). How solid is the evidence that painful experiences are damaging the brain? Indeed, in another study, pain was a pivotal predictive factor of brain dysmaturation in very preterm babies and babies exposed early to pain because they “demonstrated reduced white matter and corticospinal tract fractional anisotropy (FA) as well as lower N-acetyl-aspartate/choline in subcortical gray matter, even when comprehensively accounting for neonatal illness severity and exposure to sedatives and analgesics. Importantly, the changes in white matter FA relate to changes in diffusivity aligned along the long axis of neurons in contrast to the changes in FA related to infection and mechanical ventilation, which are perpendicular to the axonal component of diffusion. These findings further bolster the independent association of procedural pain with brain dysmaturation. The observations related to procedural pain are also congruent with studies of neonatal stress. Greater neonatal stress predicts decreased frontal and parietal brain width and altered diffusion and functional connectivity in the temporal lobes. More recent observations demonstrate that greater procedural pain, especially in early life, is associated with smaller thalamic volumes, specifically in the somatosensory thalamus, and poor functional outcomes to 3 years of age” (21).

## 4 Pain, stress, and neurodevelopment

The fetal brain is plastic and vulnerable to painful stimuli (2); this is deemed true even during the fetal-to-neonatal transition: first, prenatal and neonatal stress may disrupt and reinforce the development of the nociception system (22); second, preterm babies stimulated painfully *in utero* presented more stress hormones, a higher heart rate, and lower oxygen saturation as painful events were registered in procedural memory in a comparative study (23). Until then, the so-called fetal procedural memory was only hypothesized (24). Furthermore, the cognitive outcomes of preterm babies were worse during procedures where the integrity of the skin was compromised, and procedural pain seemed to determine an allostatic overload (25). In preterm and extremely preterm children and young adults, NICU pain experiences negatively influence neurodevelopmental outcomes, including pain response in later life (26). This is likely due to altered biological factors (i.e., peripheral and central somatosensory function and modulation, brain structure, and connectivity) and psychosocial factors (e.g., sex, coping style, mood, and parental response) (26). Additionally, we do have an anatomical and functional substrate to better understand these developmental phenomena, as a few years ago, it was not demonstrated that “early pain was associated with decreased functional connectivity between the thalami and bilateral somatosensory cortex and between the right insular cortex and ipsilateral amygdala and hippocampal regions, with a more evident effect in preterm neonates undergoing more invasive procedures. Functional connectivity of the right thalamocortical pathway was related to negative neuromotor outcomes at 24 months (*P* = 0.003). Early pain exposure is correlated with abnormal functional connectivity of developing networks involved in the modulation of noxious stimuli in preterm neonates, contributing to the neurodevelopmental consequence of preterm birth” (27); in this sense, this study was aligned with further research because an altered structural connectivity was associated with neurodevelopmental outcomes (28).

## 5 Discussion

In light of this evidence, we believe that the International Association for the Study of Pain definition of pain appears to be limited and inadequate to explain fetal-neonatal nociception and pain because noxious stimuli may result in “immediate or long-term ramification” (3), that is, a more complex network of short term and long term resulting phenomena from the initial trigger of the nociceptive experience. Thus, pain should be quantified more appropriately, perhaps with more sophisticated scales of assessment, to select the most fitting perinatal and neonatal pediatric therapy (29) synergically with neonatology, perinatology, obstetrics, fetal surgery, fetal anesthesiology, fetal neurobehavior, neuroscience, legal medicine, and medical bioethics (13). This is a tedious task, however, pain is necessary to be analyzed sociologically, and ethically, besides theology and medicine. Indeed, fetal pain implies a “patienthood”-to-“personhood” issue -, changing the concepts of maternal autonomy and pregnancy (30), and it can be conditioned by clinicians' conceptions (31), although the balance of risks and benefits between the woman and fetus is challenging (32). To better hold the patient status of fetuses, we should consider that despite they are very fragile beings who can get ill, many conditions *in utero* can influence aberrant neonatal outcomes. Therefore, as prof. Peter G. Fedor-Freybergh stated, “we need to extend the standard definition of life's continuum to include the prenatal experience, which is part of life's continuum, helping to shape us” (33).
